# Mass incarceration as a climate justice issue

**DOI:** 10.1088/1748-9326/ae5f35

**Published:** 2026-04-24

**Authors:** Katherine LeMasters, Lauren Brinkley-Rubinstein, David Ciplet, David Cloud, Kristen Cowan, William Eisenman, Lawrence Haber, Allison Macht, Colleen E Reid

**Affiliations:** 1 Division of General Internal Medicine, School of Medicine, University of Colorado Anschutz Medical Campus, Aurora, CO, United States of America; 2 Department of Epidemiology, School of Public Health, University of Colorado Anschutz Medical Campus, Aurora, CO, United States of America; 3 Department of Population Health Sciences, Duke University School of Medicine, Durham, NC, United States of America; 4 Department of Environmental Studies, University of Colorado Boulder, Boulder, CO, United States of America; 5 Department of Epidemiology, Gillings School of Global Public Health, University of North Carolina at Chapel Hill, Chapel Hill, NC, United States of America; 6 FreeWorld, San Jose, CA, United States of America; 7 Division of Hospital Medicine, Department of Medicine, Denver Health and Hospital Authority, Denver, CO, United States of America; 8 Department of Medicine, University of Colorado, Aurora, CO, United States of America; 9 Department of Geography, University of Colorado Boulder, Boulder, CO, United States of America

**Keywords:** mass incarceration, climate crisis, extreme heat, wildfire, hurricane

## Abstract

The climate crisis and mass incarceration are deeply intertwined. While climate change has intensified worldwide, incarcerated populations are disproportionately at risk of experiencing poor health related to climate change through multiple hazards including extreme heat, hurricanes, and wildfires. We detail how incarcerated individuals are at a heightened risk of experiencing multiple climate-related events, how climate change worsens the health of incarcerated individuals, and how carceral infrastructure and policies worsen these impacts. We then propose next steps including (1) further research to assess the full scope of climate-related health risks, (2) strong collaborations between researchers, policymakers, and community advocates, and (3) implementation of evidence-based policies that prioritize the well-being of incarcerated populations that span climate mitigation, climate adaptation, and decarceration measures.

## 1. Introduction

The United States is experiencing an unparalleled epidemic of mass incarceration, a system of social and racial control that affects those with criminal legal contact and entire communities where it is concentrated [1]. On a given day, the United States incarcerates two million individuals, with 1.3 million being held in prisons (state or federal facilities where individuals are often incarcerated when they receive a sentence to one or more years), seven million annually cycling through jails (county-run facilities where individuals are often held pre-trial or when sentenced for less than one year), and over 20 million adults having a history of incarceration [2]. Carceral involvement disproportionately affects racial and ethnic minorities, low-income and rural communities, sexual and gender minorities, and those with disabilities [2]. At the same time, the climate crisis is wreaking havoc on communities across the United States, with increasing heat waves, wildfires, hurricanes, and floods disproportionately impacting the very same communities most impacted by mass incarceration [3]. This unjust differential distribution of environmental health risks, largely along racial and economic lines, is referred to as climate and environmental injustice [4].

In this paper, we first argue that the climate crisis and mass incarceration are deeply intertwined, with incarcerated individuals across the United States being at a heightened risk of both exposure to climate hazards, and suffering from poor health outcomes—including death—related to climate events compared to the general population [5]. We then detail how carceral policies and infrastructure contribute to incarcerated individuals’ disproportionate exposure to and suffering from climate events. After bringing attention to these intersections, we then call for the need to develop a deeper understanding at the intersection of mass incarceration, climate hazards, and health through interdisciplinary, community-engaged research. We end by calling for the need for strong, timely policy solutions at the intersection of climate mitigation in carceral facilities, climate monitoring in carceral facilities, and decarceration, with funding contingent on facilities enacting such change. We thus address research and practice needs to reduce harm for those currently incarcerated and address the need for abolition of the system of mass incarceration in the wake of the climate crisis.

## 2. Extreme climate events: heat, wildfire, and hurricanes

People who are incarcerated suffer disproportionately from climate events due to their high exposure to multiple environmental hazards compared to the general population. This is largely related to poor conditions of confinement such as overcrowding [6], a lack of protective policies, outdated infrastructure, and the location of carceral facilities. We detail below states and regions that have experienced the most severe climate events in prisons and jails. While climate change disproportionately impacts certain regions of the United States (e.g. extreme heat in Texas prisons), it is also creating crises in areas previously thought not to be prone to such events (e.g. hurricanes in western North Carolina), highlighting the need for jails and prisons across the country to be paid attention to.

Further, counties with the highest Expected Annual Loss from climate disaster and highest incarceration rates are often overlapping—primarily counties in Texas, Florida, North Carolina, and California [7]. This clear overlap between environmental injustices and mass incarceration has led to explicit claims that prisons and jails are sites of environmental injustices [8].

For example, the prevalence of heat waves in the United States has tripled in recent years [3]. Carceral facilities are disproportionately at risk of being exposed to extreme heat as most incarcerated individuals are housed in the 44 states that do not universally provide air conditioning [9]. Facilities are also often overcrowded [10], which makes spaces hotter and stresses facility infrastructure and prevents temperature regulation [11]. Thus, prisons are increasingly experiencing days above 82.4° F indoors, the threshold for extreme heat used by the United States National Institute for Occupational Safety and Health [12]. Specifically, carceral facilities were exposed to 5.5 more days per year above 82.4° F compared to locations without carceral facilities; carceral facilities in Arizona, California, and Nevada experienced the greatest exposure disparities [12]. In California state prisons, 66% of individuals reported experiencing an extreme heat event (versus 46% of the general population), with 44% reporting fearing for their lives due to experiencing heat cramps, heat exhaustion, and heat stroke amid lack of access to air conditioning and shade [13, 14]. Heat waves are also particularly severe in prisons and jails, with indoor heat indices reaching 150°F in one Texas prison—in contrast, in California, indoor workplaces must have cooldown areas and rest breaks once indoor heat indices reach 82° F [11, 15]. Incarcerated individuals in Colorado have described this experience as ‘sitting in a convection oven being slowly cooked alive’ [16]. 3 d heat waves have resulted in an approximately 7% increase in deaths and a 15% increase in suicides in Texas state prisons [17].

Similarly, the prevalence of extreme wildfires has doubled in the past 21 years [3]. Wildfires now impact all regions of the US, often due to widespread hazardous smoke (e.g. Canadian wildfire smoke created unhealthy conditions for most United States regions in 2023) [18–20]. During the Los Angeles County wildfires in California in January 2025, over 40 carceral facilities were at risk of burning or of extreme air quality issues but those in evacuation zones did not evacuate [21]. In Colorado, nearly one-third of incarcerated individuals live in counties with a high wildfire burn probability [22]. A study conducted interviews and focus groups with previously incarcerated individuals in Colorado in 2024, finding that 37% had experienced wildfires while incarcerated [23]. Many individuals reported smoke and ash entering from cracked windows during wildfire smoke events [23]. Individuals stated ‘I could feel it. You could see it outside the window, and then of course you smell it. So, you figure it is just in there’ [23].

Heat and wildfire smoke have been shown to synergistically impact health in the general population, contributing to excess mortality [24]. This is partially due to increasing heat contributing to fire risk through drying of potential fire fuels (e.g. dead plant matter) [25], so these exposures often happen simultaneously [24]. When fires are particularly close to facilities, individuals then experience additional radiant heat, contributing to risk of heat-related illness. One incarcerated individual in Colorado noted that ‘[wildfires were] all during summer time, so it is already hot, but it feels even hotter cause you smell fire and then the ACs do not work…it is just this combination of unfortunate factors that really affect everybody’ [23]. Further, both exposures worsen mental health. Extreme heat worsens mental health symptoms by altering the body’s ability to thermoregulate and regulate emotions via disrupting sleep and neurotransmitters [26]. This leads to lethargy, irritability, sadness, and impaired cognitive functioning and decision-making. Wildfire smoke exposure then increases stress and anticipatory anxiety due to uncertainty and increases isolation as individuals cannot go outside [27].

Hurricanes also pose significant environmental hazards to incarcerated populations. Carceral facilities are often in flood-prone areas, making them vulnerable to storm surges and extreme rainfall. When hurricanes strike, incarcerated individuals frequently face minimal or no communication from the outside world and delayed or inadequate evacuation plans, with many criminal legal administrators electing to have the populations in their facilities shelter in place as surrounding communities evacuate. For example, in 2018, Hurricane Florence caused catastrophic flooding in the Carolinas, yet officials refused to evacuate several prisons in mandatory evacuation zones [28]. Similarly, in 2024, thousands of incarcerated individuals in western North Carolina were trapped in flooded cells without lights, running water, or any information for five days during Hurricane Helene [29]. These risks extend beyond immediate flooding: hurricanes can cause infrastructure failures including power outages that disrupt air circulation and supply chain disruptions that limit access to food and medical care.

## 3. How climate worsens the health of incarcerated individuals

Not only do incarcerated individuals experience heightened exposure to climate hazards compared to the general population [12, 21], but they also experience disproportionate levels of poor health impacts from exposure to such hazards. Incarcerated populations are largely comprised of subgroups that are particularly vulnerable to climate hazards, such as older adults [30], those with mental illness [31], and those with predisposing respiratory illnesses [32]. Additionally, incarcerated individuals disproportionately come from low-income and racially minoritized communities with histories of climate and environmental injustices [2, 4] and thus often have a history of harmful environmental exposures across the life course including exposure to extreme heat, lead, pollution, hurricanes and poor water quality—making these individuals even more vulnerable from suffering from such hazards during incarceration [33, 34]. For example, incarcerated individuals coming from low-income and racially minoritized communities that disproportionately experience extreme heat without adequate air conditioning likely experience worsened chronic conditions prior to incarceration, making them increasingly vulnerable to suffer from poor health during extreme heat exposure during incarceration.

First, the incarcerated population is rapidly aging, with local jails experiencing a 37% increase in the population aged 55 and older from 2020–2022, which is expected to grow in the coming years [30]. Thus, the proportion of incarcerated individuals with aging-related conditions such as dementia is increasing. Further, incarcerated individuals also experience accelerated aging—the process in which exposure to incarceration speeds up biological aging—and often have or develop serious chronic comorbidities while incarcerated (e.g. hypertension, arthritis, diabetes, heart disease, kidney problems) [5]. These conditions make individuals even more vulnerable to extreme conditions. For example, some conditions worsen with exposure to extreme temperature (e.g. heart disease), other conditions make individuals more vulnerable to harms during disasters (e.g. dementia impairs decision-making), and other conditions require medication that increase sensitivity to harmful climate exposures (e.g. antidepressants, diuretics, and beta-blockers interfere with the body’s ability to regulate temperature) [32].

Second, in the United States, 44% of those housed in county jails and 36% of those housed in state prisons have a history of mental illness, over double that of the general population [31]. Incarcerated women also have higher rates of mental illness than incarcerated men, making them disproportionately vulnerable to climate stressors [35]. This population of those with mental illness is disproportionately prescribed medications that reduce the body’s ability to thermoregulate, such as some antipsychotics and antidepressants, making them particularly vulnerable to extreme heat [36]. Individuals with pre-existing mental illness, both in prison and the general population, also face a higher risk of suicide and self-harm during extreme heat waves compared to other individuals [27].

Third, individuals with a history of incarceration have over twice the prevalence of asthma as the general population (13% vs 6%)—likely an underestimate—and are thus at particularly high risk for exacerbations of asthma during wildfire smoke events, including elevated stress and anxiety due to difficulty breathing and worsened respiratory symptoms [37]. This is only made worse by the already low access to asthma medication [38]. The incarcerated population is also at a particularly high risk of respiratory depression due to extreme cold—an increasing concern that requires further study as winter storms become more frequent and intense while carceral facilities often lack the insulation and power structures to provide sufficient heating [39].

## 4. The additive harms of carceral policies and infrastructure

Few policies protect incarcerated individuals from climate hazards. For example, during the Los Angeles area wildfires in January of 2025, no county jails responded about their plans to protect incarcerated people from air quality issues or to evacuate [40]. Additionally, many individuals incarcerated during Hurricane Katrina lived in facilities with no evacuation plan and they were left abandoned in jail and hundreds were estimated to have died [41]. Further, among all 50 state preparedness plans for climate disasters, only 30 mention incarcerated individuals, and of those plans that do mention them, it is usually only in the context of incarcerated people being part of the labor force for disaster response [42]. Only 17 state prison systems have published preparedness plans, with only six of those discussing keeping individuals safe during disasters [42].

When used as a labor force, many incarcerated people are not provided protective gear. Incarcerated firefighters are four times as likely to be injured by objects than their non-incarcerated counterparts and eight times as likely to suffer injuries related to smoke inhalation than non-incarcerated firefighters [43]. The use of incarcerated labor is included in most state-level emergency operation plans, including activities that extend across the full lifecycle of climate disasters, including mitigation, preparedness, response, and recovery [42]. As such, while many state agencies depend on the labor of incarcerated individuals as an adaptation strategy to increase societal resilience to climate change, appropriate consideration or protections are not granted to incarcerated workers.

The infrastructure of carceral facilities also amplifies environmental hazards. First, carceral facilities are disproportionately placed in environmentally degraded areas or areas with high climate disaster susceptibility [7]. At least 589 federal and state prisons are located within three miles of a superfund cleanup site (i.e. an area where hazardous waste is improperly managed), and many more are located near old mining sites and landfills [44]. Moreover, incarceration facilities are often located in isolated locations with restricted access. As a result, in addition to direct forms of climate hazard inundation and emergency evacuation procedures being particularly difficult, incarcerated populations may be more susceptible to having disrupted access to essential services, including hospitals.

Second, these facilities also have outdated infrastructure: the average age of California prisons is 45 years with multiple facilities having buildings built in the 1800’s [45], 44 state prison systems lack universal air conditioning [46], and most California prisons have lower ventilation rates than the recommended standards [45]. As previously mentioned, in prior wildfire events, individuals have reported smoke entering through cracked windows and ineffective ventilation systems [23]. Third, populations with limited mobility are disproportionately affected by environmental hazards; all incarcerated individuals have reduced mobility, with those experiencing solitary confinement having particularly reduced mobility and increased suffering from nearby wildfires (e.g. unable to go outside, reduced air conditioning flow from evaporative coolers in confined spaces). Incarcerated populations are also often unable to use adaptive measures due to limited access to cool water, fans, and other resources such as money to purchase commissary fans [11]. Furthermore, disabled and older individuals are largely unable to have jobs while incarcerated that provide resources to afford a commissary fan [27].

Lastly, the impact of a lack of protective policies for incarcerated individuals, as well as degrading physical and institutional environments, are further compounded by poor conditions of confinement, including overcrowded living conditions and a lack of preventive health measures [2]. For example, overcrowded conditions make evacuation in a disaster even more perilous and place further stress on already inadequate healthcare systems. Individuals’ deteriorating health due to lack of access to care is only worsened when individuals experience acute health-harming events such as wildfires that further exacerbate pre-existing health conditions.

## 5. Potential solutions

To begin to address the overlapping crises of mass incarceration and climate change, we must first develop a more complete understanding of the intersection of mass incarceration, climate hazards, and health. Additional research is needed into the unique health-related effects of extreme heat, extreme cold, hurricanes, flooding, wildfires, drought, and air pollution on incarcerated individuals. This work must also explore how these exposures differentially affect incarcerated populations by race, ethnicity, gender, age, mental illness, chronic conditions, and other disabilities. To do this work, we need access to real-time information on infrastructure within prisons (e.g. ventilation, air conditioning) and related risk conditions (e.g. temperature index, air quality index, flood risk), to establish interdisciplinary collaborations with engineers, architects, public health experts, climate exposure assessment experts, and litigators [47], and to collaborate with community organizations and advocates. There is also a need to employ innovative data collection methods such as open records requests and forensic architecture modeling with individuals with lived experience to push for data accountability and to produce novel data on the carceral system. We see this happening through projects such as Fight Toxic Prisons, Texas Prison Community Advocate’s 85 To Stay Alive Campaign, and The Toxic Prisons Mapping Project. The Toxic Prisons Mapping Project combines the expertise of academics, grassroots organizations, and individuals with lived experience in the carceral system to map the environmental hazards that each carceral facility across the United States faces from extreme heat, air pollution, flooding, wildfires, and more [48]. The project has found that, for example, facilities in California experience the poorest air quality and those in Arizona experience the highest temperatures.

There is also a need for strong policy solutions. We must make federal emergency management funding contingent on the adequate inclusion of carceral agencies, and their confined populations, into jurisdictions’ grant applications [42]. This also includes providing equity in safety protections for incarcerated essential workers, creating opportunities for post-incarceration employment in these sectors (e.g. firefighting), and ending forced prison labor during disasters. The Federal Prison Oversight Act, which requires transparent monitoring of federal prisons and regular facility surveillance for medical and psychiatric care, must also include climate-related monitoring [5]. The Environmental Health in Prisons Act and Declaration of Environmental Rights for Incarcerated People were introduced in 2024 by United States Senator Edward Markey and United States Congresswoman Ayanna Pressley. The act calls for climate mitigation activities (e.g. painting roofs white and upgrading or installing ventilation systems), climate monitoring (e.g. water and air quality testing), and decarceration measures because carceral facilities facilitate harm to incarcerated persons and their communities [41]. Such decarceration measures include (1) reducing prison populations, especially vulnerable populations such as older adults and those with comorbid conditions, (2) closing the most climate vulnerable carceral facilities, and (3) establishing rural-urban investments and alliances to reduce reliance on prison-related employment in rural areas disproportionately vulnerable to climate hazards [49].

## 6. Conclusion

The climate crisis disproportionately affects incarcerated populations. Due to the placement, infrastructure, and policies of carceral facilities and inequities embedded in the United States criminal legal system, incarcerated populations are increasingly exposed to environmental hazards. At the same time, incarcerated populations’ poor and deteriorating physical and mental health while incarcerated places them at particularly high risk of suffering from climate exposures compared to the general population. Ultimately, a comprehensive and urgent response is necessary to protect the health and rights of incarcerated individuals in the face of a changing climate. This requires a multi-faceted approach, including further research to assess the full scope of climate-related health risks; strong collaborations between researchers, policymakers, and community advocates; implementation of evidence-based policies that prioritize the well-being of incarcerated populations; and confronting systemic injustices endemic to systems of mass incarceration. Without meaningful action, the climate crisis will continue to exacerbate existing health inequities within carceral settings.
